# Supplementary material for: MYC Targets Scores Are Associated with Cancer Aggressiveness and Poor Survival in ER-Positive Primary and Metastatic Breast Cancer
Source: Int J Mol Sci. 2020 Oct 30;21(21):8127. doi: 10.3390/ijms21218127 (PMC7663719; doi:10.3390/ijms21218127)
Supplement: Supplementary file 1 [file ijms-21-08127-s001.pdf]

Table S1: HALLMARK_MYC_TARGETS_V1 with proliferation, survival, and positive/ negative correlation with MYC score analysis

		Proliferation correlation						Survival				Pos/Neg target	
Gene	Gene name	Spearman TCGA		Spearman METABRIC		Spearman GSE124647		COX analysis TCGA		COX analysis METABRIC		Spearman	
		R	*p* value	R	*p* value	R	*p* value	HR	*p* value	HR	*p* value	R	*p*
ABCE1	ATP binding cassette subfamily E member 1	0.357	<0.01	0.551	<0.01	0.504	<0.01	1.61	0.212	1.27	0.062	0.294	<0.01
ACP1	Acid phosphatase 1	0.496	<0.01	0.599	<0.01	0.525	<0.01	1.14	0.784	0.99	0.906	-0.024	0.56
AIMP2	aminoacyl tRNA synthetase complex interacting multifunctional protein 2	0.563	<0.01	0.483	<0.01	0.309	<0.01	2.44	0.034	1.29	0.038	0.141	<0.01
AP3S1	adaptor related protein complex 3 subunit sigma 1	0.082	0.05	0.36	<0.01	-0.163	0.05	1.73	0.056	1.05	0.376	-0.148	<0.01
APEX1	apurinic/apyrimidinic endodeoxyribonuclease 1	0.364	<0.01	0.429	<0.01	0.364	<0.01	1.12	0.81	1.2	0.071	0.095	0.02
BUB3	BUB3 mitotic checkpoint protein	0.342	<0.01	0.458	<0.01	0.354	<0.01	1.06	0.858	1.01	0.916	-0.072	0.08
C1QBP	complement C1q binding protein	0.408	<0.01	0.336	<0.01	0.481	<0.01	1.31	0.429	0.93	0.466	0.082	0.05
CAD	carbamoyl-phosphate synthetase 2, aspartate transcarbamylase, and dihydroorotase	-0.035	0.41	0.003	0.9	0.309	<0.01	1.15	0.681	1.35	0.069	0.215	<0.01
CANX	calnexin	0.283	<0.01	0.23	<0.01	0.153	0.07	1.07	0.842	1.26	0.065	-0.034	0.42
CBX3	chromobox 3	0.542	<0.01	0.052	0.05	0.42	<0.01	1.53	0.237	1.36	0.002	-0.01	0.81
CCNA2	cyclin A2	0.603	<0.01	0.647	<0.01	0.542	<0.01	1.3	0.117	1.94	<0.001	0.103	0.01
CCT2	chaperonin containing TCP1 subunit 2	0.679	<0.01	0.685	<0.01	0.529	<0.01	1.15	0.613	1.43	<0.001	0.11	<0.01
CCT3	chaperonin containing TCP1 subunit 3	0.603	<0.01	0.435	<0.01	0.435	<0.01	2.29	0.028	1.39	0.002	0.108	<0.01
CCT4	chaperonin containing TCP1 subunit 4	0.505	<0.01	-	-	0.495	<0.01	2.1	0.08	-	-	0.116	<0.01
CCT5	chaperonin containing TCP1 subunit 5	0.726	<0.01	0.299	<0.01	0.464	<0.01	1.87	0.053	1.55	<0.001	0.132	<0.01
CCT7	chaperonin containing TCP1 subunit 7	0.619	<0.01	0.583	<0.01	0.636	<0.01	1.98	0.173	1.04	0.573	0.093	0.02
CDC20	cell division cycle 20	0.593	<0.01	0.332	<0.01	0.656	<0.01	1.24	0.145	1.55	<0.001	0.031	0.46
CDC45	cell division cycle 45	0.608	<0.01	0.455	<0.01	0.452	<0.01	1.16	0.314	1.57	<0.001	0.082	0.05
CDK2	cyclin dependent kinase 2	0.368	<0.01	0.401	<0.01	0.43	<0.01	1.48	0.278	1.82	<0.001	0.005	0.9
CDK4	cyclin dependent kinase 4	0.56	<0.01	0.491	<0.01	0.403	<0.01	1.55	0.202	1.54	<0.001	-0.009	0.83
CLNS1A	chloride nucleotide-sensitive channel 1A	0.349	<0.01	0.354	<0.01	0.223	<0.01	0.97	0.912	1.2	0.003	0.048	0.25
CNBP	CCHC-type zinc finger nucleic acid binding protein	0.392	<0.01	0.442	<0.01	0.153	0.07	1.09	0.887	0.9	0.164	0.075	0.07
COPS5	COP9 signalosome subunit 5	0.598	<0.01	0.573	<0.01	0.282	<0.01	1.67	0.115	1.27	0.023	0.015	0.72
COX5A	cytochrome c oxidase subunit 5A	0.646	<0.01	0.529	<0.01	0.375	<0.01	1.1	0.804	1.25	0.1	-0.06	0.15
CSTF2	cleavage stimulation factor subunit 2	0.317	<0.01	0.247	<0.01	0.262	<0.01	2.08	0.075	1.48	0.003	-0.103	0.01
CTPS1	CTP synthase 1	-	-	-	-	0.434	<0.01	1.08	0.891	0.63	0.012		
CUL1	cullin 1	0.167	<0.01	0.052	0.06	0.487	<0.01	1.12	0.674	1.63	<0.001	0.041	0.33
CYC1	cytochrome c1	0.603	<0.01	0.464	<0.01	0.47	<0.01	1.29	0.591	1.38	0.049	0.103	0.01
DDX18	DEAD-box helicase 18	0.11	<0.01	0.08	<0.01	0.578	<0.01	1.25	0.313	1.05	0.522	0.105	0.01
DDX21	DExD-box helicase 21	0.178	<0.01	0.591	<0.01	0.339	<0.01	1.05	0.875	1.05	0.534	0.272	<0.01
DEK	DEK proto-oncogene	0.201	<0.01	0.518	<0.01	0.306	<0.01	2.32	0.032	1.02	0.852	0.155	<0.01

**DHX15**	**DEAH-box helicase 15**	0.122	<0.01	0.523	<0.01	0.277	<0.01	1.4	0.247	1.28	0.023	0	0.99
**DUT**	**deoxyuridine triphosphatase**	0.38	<0.01	0.348	<0.01	0.257	<0.01	0.66	0.247	0.88	0.034	0.167	<0.01
**EEF1B2**	**eukaryotic translation elongation factor 1 beta 2**	0.117	<0.01	0.333	<0.01	0.172	0.04	1.06	0.89	0.95	0.477	0.347	<0.01
**EIF1AX**	**eukaryotic translation initiation factor 1A X-linked**	0.203	<0.01	0.447	<0.01	0.213	0.01	1.66	0.288	-	-	0.068	0.1
**EIF2S1**	**eukaryotic translation initiation factor 2 subunit alpha**	0.406	<0.01	-	-	0.309	<0.01	1.76	0.18	1.89	<0.001	0.033	0.43
**EIF2S2**	**eukaryotic translation initiation factor 2 subunit beta**	0.604	<0.01	0.303	<0.01	0.443	<0.01	1.83	0.118	1.34	0.008	0.106	0.01
**EIF3B**	**eukaryotic translation initiation factor 3 subunit B**	0.523	<0.01	-0.052	0.06	0.491	<0.01	0.69	0.358	0.9	0.38	0.307	<0.01
**EIF3D**	**eukaryotic translation initiation factor 3 subunit D**	0.162	<0.01	-0.034	0.21	0.276	<0.01	1.59	0.368	0.88	0.33	0.306	<0.01
**EIF3J**	**eukaryotic translation initiation factor 3 subunit J**	0.453	<0.01	0.483	<0.01	0.305	<0.01	1.59	0.313	0.97	0.655	0.056	0.18
**EIF4A1**	**eukaryotic translation initiation factor 4A1**	0.471	<0.01	0.52	<0.01	-	-	1.68	0.194	0.96	0.525	0.261	<0.01
**EIF4E**	**eukaryotic translation initiation factor 4E**	0.36	<0.01	0.587	<0.01	0.294	<0.01	2.32	0.075	1.13	0.086	0.078	0.06
**EIF4G2**	**eukaryotic translation initiation factor 4 gamma 2**	0.12	<0.01	0.427	<0.01	0.061	0.47	2.19	0.196	0.93	0.252	-0.089	0.03
**EIF4H**	**eukaryotic translation initiation factor 4H**	0.14	<0.01	0.378	<0.01	0.328	<0.01	2.02	0.023	1.31	0.02	0.047	0.26
**EPRS**	**glutamyl-prolyl-tRNA synthetase**	0.254	<0.01	0.269	<0.01	0.289	<0.01	0.95	0.89	0.98	0.838	-0.018	0.67
**ERH**	**ERH mRNA splicing and mitosis factor**	0.489	<0.01	0.434	<0.01	0.326	<0.01	1.32	0.578	0.86	0.265	-0.146	<0.01
**ETF1**	**eukaryotic translation termination factor 1**	0.238	<0.01	0.5	<0.01	0.085	0.32	1.37	0.428	1.07	0.682	-0.039	0.35
**EXOSC7**	**exosome component 7**	0.321	<0.01	0.044	0.1	0.32	<0.01	0.59	0.294	0.76	0.053	0.048	0.25
**FAM120A**	**family with sequence similarity 120A**	-0.059	0.15	-0.026	0.34	-0.21	0.01	0.84	0.595	1.03	0.815	-0.083	0.05
**FBL**	**fibrillarin**	0.303	<0.01	0.191	<0.01	0.461	<0.01	1.23	0.596	0.95	0.556	0.321	<0.01
**G3BP1**	**G3BP stress granule assembly factor 1**	0.261	<0.01	0.386	<0.01	0.206	0.01	0.73	0.369	1.17	0.074	0.009	0.83
**GLO1**	**glyoxalase I**	0.51	<0.01	0.474	<0.01	0.287	<0.01	1.74	0.165	0.95	0.626	0.009	0.83
**GNL3**	**G protein nucleolar 3**	0.359	<0.01	0.498	<0.01	-	-	1.66	0.079	1.14	0.117	0.286	<0.01
**GOT2**	**glutamic-oxaloacetic transaminase 2**	0.407	<0.01	0.021	0.45	0.165	0.05	0.98	0.954	1.34	0.027	0	1
**GSPT1**	**G1 to S phase transition 1**	0.285	<0.01	0.249	<0.01	0.009	0.91	1.54	0.166	1.53	<0.001	-0.09	0.03
**H2AFZ**	**H2A histone family, member Z**	0.684	<0.01	0.706	<0.01	0.557	<0.01	2.69	0.072	1.89	<0.001	-0.005	0.91
**HDAC2**	**histone deacetylase 2**	0.358	<0.01	0.458	<0.01	0.383	<0.01	1.57	0.083	1.22	0.032	0.012	0.77
**HDDC2**	**HD domain containing 2**	0.25	<0.01	0.117	<0.01	0.122	0.15	1.2	0.581	1.01	0.944	-0.037	0.37
**HDGF**	**heparin binding growth factor**	0.446	<0.01	0.381	<0.01	0.222	<0.01	1.01	0.975	1.41	0.007	0.084	0.04
**HNRNPA1**	**heterogeneous nuclear ribonucleoprotein A1**	0.275	<0.01	0.471	<0.01	0.188	0.03	0.7	0.406	0.93	0.233	0.135	<0.01
**HNRNPA2B1**	**Heterogeneous Nuclear Ribonucleoprotein A2/B1**	0.476	<0.01	-0.183	<0.01	0.528	<0.01	2.43	0.109	1	0.98	-0.085	0.04
**HNRNPA3**	**heterogeneous nuclear ribonucleoprotein A3**	0.268	<0.01	0.239	<0.01	0.164	0.05	0.49	0.247	0.72	0.006	0.023	0.58
**HNRNPC**	**heterogeneous nuclear ribonucleoprotein C**	0.516	<0.01	-	-	0.431	<0.01	1.54	0.487	-	-	-0.09	0.03
**HNRNPCL1**		-	-	0.041	0.13	-	-	-	-	0.78	0.508	-0.048	0.08
**HNRNPD**	**heterogeneous nuclear ribonucleoprotein D**	0.327	<0.01	0.351	<0.01	0.262	<0.01	0.76	0.576	1.45	0.01	0.106	0.01
**HNRNPR**	**heterogeneous nuclear ribonucleoprotein R**	0.271	<0.01	0.269	<0.01	0.351	<0.01	0.85	0.785	1.12	0.424	0.116	<0.01
**HNRNPU**	**heterogeneous nuclear ribonucleoprotein U**	0.27	<0.01	-0.094	<0.01	0.227	<0.01	0.83	0.776	0.92	0.424	-0.162	<0.01

**HPRT1**	**hypoxanthine phosphoribosyltransferase 1**	0.6	<0.01	0.599	<0.01	0.402	<0.01	1.27	0.461	1.29	0.005	0.042	0.31
**HSP90AB1**	**heat shock protein 90 alpha family class B member 1**	0.642	<0.01	0.371	<0.01	0.513	<0.01	1.91	0.059	1.67	<0.001	0.164	<0.01
**HSPD1**	**heat shock protein family D (Hsp60) member 1**	0.744	<0.01	0.728	<0.01	0.676	<0.01	1.76	0.041	1.3	0.002	0.198	<0.01
**HSPE1**	**heat shock protein family E (Hsp10) member 1**	0.643	<0.01	-	-	0.541	<0.01	1.59	0.09	-	-	-0.136	<0.01
**IARS**	**Isoleucyl-TRNA Synthetase 1**	0.275	<0.01	0.519	<0.01	0.333	<0.01	1.66	0.117	1.22	0.056	0.108	<0.01
**IFRD1**	**interferon related developmental regulator 1**	-0.07	0.09	0.063	0.02	0.113	0.18	1.36	0.18	0.9	0.416	0.204	<0.01
**ILF2**	**interleukin enhancer binding factor 2**	0.454	<0.01	0.488	<0.01	0.518	<0.01	1.65	0.171	1.19	0.099	-0.066	0.11
**IMPDH2**	**inosine monophosphate dehydrogenase 2**	0.242	<0.01	0.126	<0.01	0.467	<0.01	0.86	0.61	0.76	0.002	0.316	<0.01
**KARS**	**Lysyl-TRNA Synthetase 1**	0.456	<0.01	0.331	<0.01	0.179	0.03	1.87	0.111	1.46	0.004	0.14	<0.01
**KPNA2**	**karyopherin subunit alpha 2**	0.661	<0.01	0.751	<0.01	0.505	<0.01	1.28	0.215	1.22	0.001	0.003	0.94
**KPNB1**	**karyopherin subunit beta 1**	0.317	<0.01	0.424	<0.01	0.07	0.41	0.77	0.54	0.86	0.263	0.076	0.07
**LDHA**	**lactate dehydrogenase A**	0.465	<0.01	0.418	<0.01	0.007	0.93	1.18	0.61	1.24	0.03	-0.031	0.46
**LSM2.00**	**LSM2 homolog, U6 small nuclear RNA and mRNA degradation associated**	0.522	<0.01	0.173	<0.01	0.501	<0.01	0.85	0.656	1.44	0.02	0.042	0.31
**LSM7.00**	**LSM7 homolog, U6 small nuclear RNA and mRNA degradation associated**	0.162	<0.01	0.446	<0.01	0.408	<0.01	1.16	0.545	1.1	0.501	0.063	0.13
**MAD2L1**	**mitotic arrest deficient 2 like 1**	0.647	<0.01	0.804	<0.01	0.598	<0.01	1.19	0.364	1.39	<0.001	0.14	<0.01
**MCM2**	**minichromosome maintenance complex component 2**	0.528	<0.01	0.362	<0.01	0.558	<0.01	1.07	0.776	1.74	<0.001	0.109	<0.01
**MCM4**	**minichromosome maintenance complex component 4**	0.483	<0.01	0.418	<0.01	0.631	<0.01	1.43	0.081	1.46	<0.001	0.092	0.03
**MCM5**	**minichromosome maintenance complex component 5**	0.254	<0.01	0.082	<0.01	0.402	<0.01	0.73	0.287	1.49	<0.001	0.133	<0.01
**MCM6**	**minichromosome maintenance complex component 6**	0.457	<0.01	0.384	<0.01	0.624	<0.01	1.31	0.34	1.79	<0.001	0.022	0.6
**MCM7**	**minichromosome maintenance complex component 7**	0.429	<0.01	0.296	<0.01	0.599	<0.01	1.1	0.723	1.45	<0.001	0.213	<0.01
**MRPL23**	**mitochondrial ribosomal protein L23**	0.282	<0.01	0.119	<0.01	0.356	<0.01	0.9	0.668	1.29	0.047	0.015	0.71
**MRPL9**	**mitochondrial ribosomal protein L9**	0.211	<0.01	0.349	<0.01	0.211	0.01	1.12	0.783	1.27	0.087	-0.008	0.84
**MRPS18B**	**mitochondrial ribosomal protein S18B**	0.469	<0.01	0.317	<0.01	0.319	<0.01	0.87	0.744	0.77	0.076	0.049	0.24
**MYC**	**MYC proto-oncogene, bHLH transcription factor**	0.126	<0.01	0.429	<0.01	0.471	<0.01	0.82	0.246	1.02	0.634	1	<0.01
**NAP1L1**	**nucleosome assembly protein 1 like 1**	0.366	<0.01	0.387	<0.01	0.185	0.03	0.54	0.174	1.03	0.78	0.279	<0.01
**NCBP1**	**nuclear cap binding protein subunit 1**	0.186	<0.01	0.131	<0.01	0.204	0.02	1.98	0.095	1.57	0.001	-0.054	0.19
**NCBP2**	**nuclear cap binding protein subunit 2**	0.44	<0.01	0.373	<0.01	0.383	<0.01	0.94	0.911	1.71	<0.001	0.068	0.1
**NDUFAB1**	**NADH:ubiquinone oxidoreductase subunit AB1**	0.589	<0.01	0.522	<0.01	0.194	0.02	1.29	0.502	0.99	0.953	-0.131	<0.01
**NHP2**	**NHP2 ribonucleoprotein**	0.508	<0.01	0.548	<0.01	0.48	<0.01	1.26	0.491	1.27	0.027	0.145	<0.01
**NME1**	**NME/NM23 nucleoside diphosphate kinase 1**	0.673	<0.01	0.594	<0.01	0.486	<0.01	0.87	0.559	1.32	<0.001	0.109	<0.01
**NOLC1**	**nucleolar and coiled-body phosphoprotein 1**	0.466	<0.01	0.531	<0.01	0.535	<0.01	1.72	0.183	1.38	0.008	0.302	<0.01
**NOP16**	**NOP16 nucleolar protein**	0.559	<0.01	0.362	<0.01	0.422	<0.01	1.52	0.166	1.73	<0.001	0.259	<0.01
**NOP56**	**NOP56 ribonucleoprotein**	0.546	<0.01	0.293	<0.01	-	-	1.56	0.188	1.61	<0.001	0.311	<0.01
**NPM1**	**nucleophosmin 1**	0.581	<0.01	-	-	0.473	<0.01	1.17	0.718	-	-	0.151	<0.01
**ODC1**	**ornithine decarboxylase 1**	0.127	<0.01	0.174	<0.01	0.156	0.07	0.98	0.929	1.11	0.161	0.297	<0.01

ORC2	origin recognition complex subunit 2	-	-	0.068	0.01	0.348	<0.01	-	-	0.96	0.85	-0.125	<0.01
ORC2L	Origin Recognition Complex Subunit 2	-0.064	0.13	-	-	-	-	0.88	0.78	-	-	0.035	0.39
PA2G4	proliferation-associated 2G4	0.733	<0.01	0.143	<0.01	0.543	<0.01	1.7	0.223	1.89	<0.001	0.06	0.15
PABPC1	poly(A) binding protein cytoplasmic 1	0.441	<0.01	0.413	<0.01	0.3	<0.01	1.32	0.287	0.95	0.393	0.342	<0.01
PABPC4	poly(A) binding protein cytoplasmic 4	0.119	<0.01	0.069	0.01	-	-	1.68	0.152	1.16	0.165	0.318	<0.01
PCBP1	poly(rC) binding protein 1	0.242	<0.01	0.18	<0.01	-0.001	0.99	1.3	0.669	1	0.978	0.016	0.69
PCNA	proliferating cell nuclear antigen	0.617	<0.01	0.654	<0.01	0.382	<0.01	1.36	0.247	1.16	0.026	-0.039	0.35
PGK1.00	phosphoglycerate kinase 1	0.487	<0.01	0.573	<0.01	0.295	<0.01	2.99	<0.001	1.11	0.081	-0.002	0.96
PHB	prohibitin	0.489	<0.01	0.243	<0.01	0.364	<0.01	0.79	0.524	1.3	0.009	0.17	<0.01
PHB2	prohibitin 2	0.339	<0.01	0.341	<0.01	0.372	<0.01	1.08	0.856	0.84	0.224	0.173	<0.01
POLD2	DNA polymerase delta 2, accessory subunit	0.571	<0.01	0.175	<0.01	0.555	<0.01	1.57	0.162	1.44	0.001	0.19	<0.01
POLE3	DNA polymerase epsilon 3, accessory subunit	0.381	<0.01	0.38	<0.01	0.418	<0.01	0.98	0.973	1.35	0.038	-0.029	0.49
PPIA	peptidylprolyl isomerase A	0.692	<0.01	0.625	<0.01	0.223	<0.01	1.65	0.153	0.96	0.433	0.072	0.09
PPM1G	protein phosphatase, Mg2+/Mn2+ dependent 1G	0.543	<0.01	0.081	<0.01	0.397	<0.01	1.26	0.591	1.98	<0.001	0.015	0.71
PRDX3	peroxiredoxin 3	0.25	<0.01	0.414	<0.01	0.049	0.56	2.15	0.064	0.93	0.114	-0.038	0.36
PRDX4	peroxiredoxin 4	0.575	<0.01	0.485	<0.01	0.225	<0.01	2.26	0.008	1.09	0.364	0.14	<0.01
PRPF31	pre-mRNA processing factor 31	0.152	<0.01	0.134	<0.01	0.156	0.07	1.39	0.288	1.36	0.017	0.081	0.05
PRPS2	phosphoribosyl pyrophosphate synthetase 2	0.243	<0.01	0.322	<0.01	0.272	<0.01	1.15	0.628	0.91	0.385	0.042	0.31
PSMA1	proteasome 20S subunit alpha 1	0.565	<0.01	0.563	<0.01	0.07	0.41	1.31	0.53	1.1	0.294	-0.068	0.1
PSMA2	proteasome 20S subunit alpha 2	0.561	<0.01	0.371	<0.01	0.28	<0.01	2.72	0.022	0.9	0.422	-0.137	<0.01
PSMA4	proteasome 20S subunit alpha 4	0.534	<0.01	0.496	<0.01	0.224	<0.01	1.27	0.499	1.07	0.535	-0.151	<0.01
PSMA6	proteasome 20S subunit alpha 6	0.624	<0.01	0.674	<0.01	-	-	1.26	0.518	1.16	0.051	-0.17	<0.01
PSMA7	proteasome 20S subunit alpha 7	0.525	<0.01	0.161	<0.01	0.464	<0.01	1.37	0.244	2.02	0.008	-0.005	0.9
PSMB2	proteasome 20S subunit beta 2	0.426	<0.01	0.369	<0.01	-0.138	0.1	1.61	0.259	1.49	0.001	-0.1	0.02
PSMB3	proteasome 20S subunit beta 3	0.416	<0.01	0.218	<0.01	0.11	0.19	0.75	0.394	1.38	0.01	-0.085	0.04
PSMC4	proteasome 26S subunit, ATPase 4	0.508	<0.01	0.449	<0.01	0.337	<0.01	0.88	0.733	1.1	0.19	-0.173	<0.01
PSMC6	proteasome 26S subunit, ATPase 6	0.472	<0.01	0.53	<0.01	0.253	<0.01	1.76	0.183	1.12	0.22	-0.168	<0.01
PSMD1	proteasome 26S subunit, non-ATPase 1	0.348	<0.01	0.293	<0.01	0.152	0.07	1.81	0.186	1.38	0.019	-0.081	0.05
PSMD14	proteasome 26S subunit, non-ATPase 14	0.595	<0.01	0.659	<0.01	0.399	<0.01	2.75	0.007	1.14	0.177	-0.153	<0.01
PSMD3	proteasome 26S subunit, non-ATPase 3	0.415	<0.01	0.164	<0.01	0.272	<0.01	1.29	0.451	1.27	0.092	0.061	0.14
PSMD7	proteasome 26S subunit, non-ATPase 7	0.413	<0.01	0.499	<0.01	0.067	0.43	2.51	0.004	1.14	0.104	0.062	0.14
PSMD8	proteasome 26S subunit, non-ATPase 8	0.545	<0.01	0.014	0.62	0.348	<0.01	1.43	0.429	1.01	0.949	-0.053	0.2
PTGES3	prostaglandin E synthase 3	0.659	<0.01	0.573	<0.01	0.406	<0.01	2.53	0.037	1.03	0.585	-0.056	0.18
PWP1	PWP1 homolog, endonuclein	0.475	<0.01	0.479	<0.01	0.257	<0.01	1.41	0.509	1.6	0.005	0.075	0.07
RAD23B	RAD23 homolog B, nucleotide excision repair protein	0.307	<0.01	0.244	<0.01	0.028	0.74	2.27	0.089	1.12	0.371	-0.121	<0.01
RAN	RAN, member RAS oncogene family	0.754	<0.01	0.63	<0.01	0.603	<0.01	1.3	0.479	1.14	0.173	0.151	<0.01
RANBP1	RAN binding protein 1	0.609	<0.01	0.613	<0.01	0.388	<0.01	0.8	0.579	1.14	0.145	0.185	<0.01
RFC4	replication factor C subunit 4	0.554	<0.01	0.68	<0.01	0.347	<0.01	1.05	0.88	1.55	<0.001	-0.007	0.86

**RNPS1**	**RNA binding protein with serine rich domain 1**	0.321	<0.01	0.254	<0.01	0.369	<0.01	1.18	0.68	1.1	0.26	0.023	0.58
**RPL14**	**ribosomal protein L14**	0.243	<0.01	0.182	<0.01	0.329	<0.01	0.72	0.265	1.01	0.896	0.2	<0.01
**RPL18**	**ribosomal protein L18**	0.165	<0.01	0.328	<0.01	0.041	0.63	0.92	0.735	0.93	0.382	0.187	<0.01
**RPL22**	**ribosomal protein L22**	0.169	<0.01	0.258	<0.01	0.208	0.01	0.58	0.171	0.83	0.006	0.24	<0.01
**RPL34**	**ribosomal protein L34**	0.025	0.55	0.623	<0.01	0.103	0.22	0.73	0.233	1.23	0.014	0.162	<0.01
**RPLP0**	**ribosomal protein lateral stalk subunit P0**	0.339	<0.01	0.587	<0.01	0.344	<0.01	1.2	0.575	0.98	0.735	0.303	<0.01
**RPS10**	**ribosomal protein S10**	0.265	<0.01	0.23	<0.01	0.103	0.23	0.81	0.501	1.08	0.575	0.243	<0.01
**RPS2**	**ribosomal protein S2**	0.322	<0.01	0.328	<0.01	-	-	1.1	0.723	0.95	0.513	0.189	<0.01
**RPS3**	**ribosomal protein S3**	0.087	0.04	0.318	<0.01	0.121	0.16	0.75	0.292	1.01	0.893	0.236	<0.01
**RPS5**	**ribosomal protein S5**	0.232	<0.01	0.552	<0.01	0.423	<0.01	1.06	0.853	0.93	0.332	0.495	<0.01
**RPS6**	**ribosomal protein S6**	0.066	0.11	0.315	<0.01	0.106	0.21	0.83	0.498	0.77	0.002	0.262	<0.01
**RRM1**	**ribonucleotide reductase catalytic subunit M1**	0.423	<0.01	0.228	<0.01	0.314	<0.01	1.08	0.821	1.55	<0.001	-0.018	0.67
**RRP9**	**ribosomal RNA processing 9, U3 small nucleolar RNA binding protein**	0.328	<0.01	0.207	<0.01	0.456	<0.01	1.09	0.8	1.2	0.393	0.231	<0.01
**RSL1D1**	**ribosomal L1 domain containing 1**	0.367	<0.01	0.193	<0.01	0.272	<0.01	0.91	0.844	0.83	0.052	0.247	<0.01
**RUVBL2**	**RuvB like AAA ATPase 2**	0.482	<0.01	0.373	<0.01	0.515	<0.01	1.22	0.499	1.71	<0.001	0.063	0.13
**SERBP1**	**SERPINE1 mRNA binding protein 1**	0.239	<0.01	-0.075	<0.01	0.462	<0.01	0.87	0.757	1.05	0.717	0.263	<0.01
**SET**	**SET nuclear proto-oncogene**	0.49	<0.01	0.373	<0.01	0.309	<0.01	1.38	0.542	1.3	0.012	0.089	0.03
**SF3A1**	**splicing factor 3a subunit 1**	-0.133	<0.01	0.162	<0.01	0.1	0.24	0.32	0.009	0.88	0.384	0.189	<0.01
**SF3B3**	**splicing factor 3b subunit 3**	0.282	<0.01	-0.006	0.82	0.203	0.02	1.96	0.038	1.59	<0.001	0.205	<0.01
**SLC25A3**	**solute carrier family 25 member 3**	0.484	<0.01	0.392	<0.01	0.344	<0.01	1.48	0.4	1.17	0.283	0.11	<0.01
**SMARCC1**	**SWI/SNF related, matrix associated, actin dependent regulator of chromatin subfamily c member 1**	0.193	<0.01	0.355	<0.01	0.368	<0.01	1.06	0.876	0.95	0.663	0.169	<0.01
**SNRPA**	**small nuclear ribonucleoprotein polypeptide A**	0.419	<0.01	0.027	0.32	0.423	<0.01	0.85	0.665	1.57	0.001	0.145	<0.01
**SNRPA1**	**small nuclear ribonucleoprotein polypeptide A'**	0.607	<0.01	0.457	<0.01	0.537	<0.01	1.29	0.318	1.51	<0.001	-0.018	0.67
**SNRPB2**	**small nuclear ribonucleoprotein polypeptide B2**	0.486	<0.01	0.391	<0.01	0.136	0.11	1.62	0.083	1.77	<0.001	-0.104	0.01
**SNRPD1**	**small nuclear ribonucleoprotein D1 polypeptide**	0.702	<0.01	0.624	<0.01	0.644	<0.01	1.56	0.218	1.33	0.034	0.118	<0.01
**SNRPD2**	**small nuclear ribonucleoprotein D2 polypeptide**	0.504	<0.01	0.433	<0.01	0.527	<0.01	0.97	0.923	1.46	0.009	0.02	0.63
**SNRPD3**	**small nuclear ribonucleoprotein D3 polypeptide**	0.484	<0.01	-	-	0.391	<0.01	1.07	0.863	-	-	0.004	0.92
**SNRPG**	**small nuclear ribonucleoprotein polypeptide G**	0.569	<0.01	0.412	<0.01	0.509	<0.01	1.53	0.218	1.27	0.056	-0.212	<0.01
**SRM**	**spermidine synthase**	0.304	<0.01	0.044	0.1	0.407	<0.01	1.49	0.128	1.16	0.124	0.201	<0.01
**SRPK1**	**SRSF protein kinase 1**	0.388	<0.01	0.453	<0.01	0.122	0.15	1.35	0.323	1.73	<0.001	0.183	<0.01
**SRSF1**	**serine and arginine rich splicing factor 1**	-	-	0.262	<0.01	0.437	<0.01	-	-	1.28	0.074	-0.21	<0.01
**SRSF2**	**serine and arginine rich splicing factor 2**	-	-	0.455	<0.01	-	-	-	-	1.57	0.002	0.223	<0.01
**SRSF3**	**serine and arginine rich splicing factor 3**	-	-	0.481	<0.01	0.594	<0.01	-	-	0.86	0.246	0.221	<0.01

**SRSF7**	**serine and arginine rich splicing factor 7**	-	-	0.237	<0.01	0.221	<0.01	-	-	0.9	0.437	0.137	<0.01
**SSB**	**small RNA binding exonuclease protection factor La**	0.516	<0.01	0.524	<0.01	0.419	<0.01	2.02	0.184	1.01	0.912	0.05	0.23
**SSBP1**	**single stranded DNA binding protein 1**	0.489	<0.01	0.558	<0.01	0.086	0.31	1.26	0.567	0.82	0.074	-0.16	<0.01
**STARD7**	**StAR related lipid transfer domain containing 7**	0.361	<0.01	0.484	<0.01	0.159	0.06	0.72	0.505	0.97	0.765	0.072	0.08
**SYNCRIP**	**synaptotagmin binding cytoplasmic RNA interacting protein**	0.176	<0.01	0.475	<0.01	0.433	<0.01	1.9	0.084	1.21	0.056	0.041	0.32
**TARDBP**	**TAR DNA binding protein**	0.463	<0.01	-0.128	<0.01	0.288	<0.01	2.07	0.481	0.96	0.802	-0.012	0.77
**TCP1**	**t-complex 1**	0.506	<0.01	0.592	<0.01	-	-	2.41	0.001	1.08	0.313	0.01	0.81
**TFDP1**	**transcription factor Dp-1**	0.14	<0.01	0.358	<0.01	0.269	<0.01	0.94	0.868	1.09	0.394	0.43	0.104
**TOMM70A**	**translocase of outer mitochondrial membrane 70**	0.434	<0.01	0.326	<0.01	0.198	0.02	1.51	0.324	1.34	0.056	0.07	0.09
**TRA2B**	**transformer 2 beta homolog**	0.415	<0.01	0.259	<0.01	0.571	<0.01	1.28	0.731	1.07	0.604	0.084	0.04
**TRIM28**	**tripartite motif containing 28**	0.323	<0.01	0.048	0.08	0.486	<0.01	1.14	0.732	1.37	0.003	0.096	0.02
**TUFM**	**Tu translation elongation factor, mitochondrial**	0.331	<0.01	0.044	0.1	0.26	<0.01	1.04	0.922	1.28	0.056	-0.039	0.35
**TXNL4A**	**thioredoxin like 4A**	0.589	<0.01	-	-	0.318	<0.01	1.93	0.032	-	-	0.111	<0.01
**TYMS**	**thymidylate synthetase**	0.557	<0.01	0.631	<0.01	0.387	<0.01	1.31	0.174	1.47	<0.001	0.103	0.01
**U2AF1**	**U2 small nuclear RNA auxiliary factor 1**	0.228	<0.01	0.102	<0.01	0.381	<0.01	1.11	0.784	1.36	0.015	-0.05	0.23
**UBA2**	**ubiquitin like modifier activating enzyme 2**	0.428	<0.01	0.331	<0.01	0.388	<0.01	1.18	0.682	0.9	0.667	0.198	<0.01
**UBE2E1**	**ubiquitin conjugating enzyme E2 E1**	0.324	<0.01	0.409	<0.01	0.232	<0.01	2.48	0.058	0.96	0.606	0.02	0.63
**UBE2L3**	**ubiquitin conjugating enzyme E2 L3**	0.393	<0.01	-0.125	<0.01	0.232	<0.01	0.56	0.253	1.25	0.019	-0.011	0.8
**USP1**	**ubiquitin specific peptidase 1**	0.232	<0.01	0.504	<0.01	0.462	<0.01	0.76	0.378	1.06	0.578	0.084	0.04
**VBP1**	**VHL binding protein 1**	0.464	<0.01	0.611	<0.01	0.221	<0.01	2.19	0.04	1.02	0.827	-0.062	0.14
**VDAC1**	**voltage dependent anion channel 1**	0.533	<0.01	0.496	<0.01	0.196	0.02	2.66	0.009	1.14	0.213	-0.063	0.13
**VDAC3**	**voltage dependent anion channel 3**	0.523	<0.01	0.421	<0.01	0.148	0.08	0.73	0.295	1.28	0.012	0.001	0.99
**XPO1**	**exportin 1**	0.211	<0.01	0.323	<0.01	0.368	<0.01	1.62	0.265	1.5	0.013	0.014	0.74
**XPOT**	**exportin for tRNA**	0.457	<0.01	0.566	<0.01	0.415	<0.01	1.49	0.105	1.7	<0.001	0.081	0.05
**XRCC6**	**X-ray repair cross complementing 6**	0.424	<0.01	0.41	<0.01	0.197	0.02	0.53	0.173	0.98	0.812	0.146	<0.01
**YWHAE**	**tyrosine 3-monooxygenase/tryptophan 5-monooxygenase activation protein epsilon**	0.264	<0.01	-0.048	0.08	0.346	<0.01	1.34	0.512	1.01	0.948	-0.11	<0.01
**YWHAQ**	**tyrosine 3-monooxygenase/tryptophan 5-monooxygenase activation protein theta**	0.338	<0.01	0.431	<0.01	0.281	<0.01	1.2	0.609	1.21	0.088	0.105	0.01

Note: For each gene correlation was performed with proliferation score in all cohorts and R value as well as *p* value are shown. COX analysis was performed to examine survival difference in TCGA and METABRIC cohorts and hazard ratio (HR) and *p* value are shown for each gene. Correlation analysis was peformed for each gene using TCGA cohort in order to quantify the positive or negative impact of each target gene on the overall MYC score. Spearman R value and *p* value for each gene is shown. Of note, gene information was not available in TCGA for the following genes and METABRIC data was used instead: HNRNPCL1, ORC2, RPS5, SRSF1, SRSF2, SRSF3, SRSF7.

Table S2: HALLMARK_MYC_TARGETS_V2 with proliferation, survival, and positive/ negative correlation with MYC score analysis

		Proliferation correlation						Survival analysis				Pos/neg targets	
Gene	Gene name	Spearman		Spearman		Spearman		COX		COX		Spearman	
		R	*p* value	R	*p* value	R	*p* value	HR	*p* value	HR	*p* value	R	p value
**AIMP2**	**aminoacyl tRNA synthetase complex interacting multifunctional protein 2**	0.467	<0.01	0.624	<0.01	0.48	<0.01	2.44	0.034	1.29	0.038	0.141	<0.01
**BYSL**	**bystin like**	0.459	<0.01	0.732	<0.01	0.724	<0.01	1.49	0.208	1.69	<0.001	0.392	<0.01
**CBX3**	**chromobox 3**	0.194	0.02	0.385	<0.01	0.353	<0.01	1.53	0.237	1.36	0.002	-0.01	0.81
**CDK4**	**cyclin dependent kinase 4**	0.322	<0.01	0.548	<0.01	0.358	<0.01	1.55	0.202	1.54	<0.001	-0.009	0.83
**DCTPP1**	**Deoxycytidine-Triphosphatase 1**	0.379	<0.01	0.453	<0.01	0.187	<0.01	1.64	0.098	0.98	0.792	-0.02	0.63
**DDX18**	**DEAD-box helicase 18**	0.586	<0.01	-0.035	0.4	0.219	<0.01	1.29	0.591	1.38	0.049	0.105	0.01
**DUSP2**	**dual specificity phosphatase 2**	0.29	<0.01	0.109	<0.01	0.151	<0.01	0.83	0.245	1.3	0.089	0.363	<0.01
**EXOSC5**	**Exosome Component 5**	0.518	<0.01	0.572	<0.01	0.559	<0.01	0.83	0.536	1.39	0.011	0.256	<0.01
**FARSA**	**phenylalanyl-tRNA synthetase subunit alpha**	0.489	<0.01	0.553	<0.01	0.562	<0.01	2.46	0.016	1.43	0.004	0.14	<0.01
**GNL3**	**G protein nucleolar 3**	-	-	0.387	<0.01	0.25	<0.01	1.74	0.165	0.95	0.626	0.286	<0.01
**GRWD1**	**glutamate rich WD repeat containing 1**	0.224	<0.01	0.503	<0.01	0.404	<0.01	1.22	0.641	1.32	0.101	0.184	<0.01
**HK2**	**hexokinase 2**	0.214	0.01	0.092	0.03	0.055	0.04	1.13	0.559	1.03	0.674	0.008	0.85
**HSPD1**	**heat shock protein family D (Hsp60) member 1**	0.713	<0.01	0.633	<0.01	0.451	<0.01	1.76	0.041	1.3	0.002	0.198	<0.01
**HSPE1**	**heat shock protein family E (Hsp10) member 1**	0.52	<0.01	0.556	<0.01	-	-	1.59	0.09	-	-	-0.136	<0.01
**IMP4**	**IMP U3 small nucleolar ribonucleoprotein 4**	0.495	<0.01	0.495	<0.01	0.275	<0.01	1.71	0.205	1.14	0.338	0.036	0.39
**IPO4**	**importin 4**	0.631	<0.01	0.584	<0.01	0.596	<0.01	1.73	0.112	1.68	<0.001	0.283	<0.01
**LAS1L**	**LAS1 like ribosome biogenesis factor**	0.559	<0.01	0.535	<0.01	0.487	<0.01	1.31	0.557	1.51	0.002	-0.015	0.72
**MAP3K6**	**mitogen-activated protein kinase kinase kinase 6**	0.274	<0.01	-0.189	<0.01	-0.197	<0.01	0.95	0.81	0.71	<0.001	0.027	0.52
**MCM4**	**minichromosome maintenance complex component 4**	0.65	<0.01	0.428	<0.01	0.633	<0.01	1.43	0.081	1.46	<0.001	0.092	0.03
**MCM5**	**minichromosome maintenance complex component 5**	0.436	<0.01	0.354	<0.01	0.365	<0.01	0.73	0.287	1.49	<0.001	0.133	<0.01
**MPHOSPH1**	**M-phase phosphoprotein 10**	0.397	<0.01	0.182	<0.01	0.218	<0.01	2.06	0.086	1.24	0.163	-0.06	0.15
**MRTO4**	**MRT4 homolog, ribosome maturation factor**	0.634	<0.01	0.61	<0.01	0.542	<0.01	1.1	0.806	1.29	0.067	0.271	<0.01
**MYBBP1A**	**MYB binding protein 1a**	0.42	<0.01	0.359	<0.01	0.301	<0.01	0.97	0.929	0.98	0.932	0.292	<0.01
**MYC**	**MYC proto-oncogene, bHLH transcription factor**	0.501	<0.01	0.291	<0.01	0.319	<0.01	0.82	0.246	1.02	0.634	1	<0.01

**NDUFAF4**	**NADH:ubiquinone oxidoreductase complex assembly factor 4**	0.306	<0.01	0.341	<0.01	0.115	<0.01	1.24	0.468	1.29	0.028	0.189	<0.01
**NIP7**	**nucleolar pre-rRNA processing protein NIP7**	0.324	<0.01	0.344	<0.01	0.299	<0.01	1.41	0.356	1.22	0.092	0.233	<0.01
**NOC4L**	**nucleolar complex associated 4 homolog**	0.442	<0.01	0.452	<0.01	0.364	<0.01	1.01	0.98	1.5	0.01	0.059	0.15
**NOLC1**	**nucleolar and coiled-body phosphoprotein 1**	0.619	<0.01	0.479	<0.01	0.532	<0.01	1.72	0.183	1.38	0.008	0.302	<0.01
**NOP16**	**NOP16 nucleolar protein**	0.56	<0.01	0.713	<0.01	0.694	<0.01	1.52	0.166	1.73	<0.001	0.259	<0.01
**NOP2**	**NOP2 nucleolar protein**	0.373	<0.01	0.641	<0.01	0.582	<0.01	1.1	0.775	1.66	<0.001	0.202	<0.01
**NOP56**	**NOP56 ribonucleoprotein**	-	-	0.68	<0.01	0.553	<0.01	1.56	0.188	1.61	<0.001	0.311	<0.01
**NPM1**	**nucleophosmin 1**	0.416	<0.01	0.409	<0.01	-	-	1.17	0.718	-	-	0.015	<0.01
**PA2G4**	**proliferation-associated 2G4**	0.462	<0.01	0.664	<0.01	0.596	<0.01	1.7	0.223	1.89	<0.001	0.06	0.15
**PES1.00**	**pescadillo ribosomal biogenesis factor 1**	0.671	<0.01	0.541	<0.01	0.331	<0.01	0.75	0.416	1.27	0.239	0.286	<0.01
**PHB**	**prohibitin**	0.419	<0.01	0.591	<0.01	0.388	<0.01	0.79	0.524	1.3	0.009	0.17	<0.01
**PLK1**	**polo like kinase 1**	0.405	<0.01	0.586	<0.01	0.579	<0.01	1.36	0.048	3.33	<0.001	0.065	0.12
**PLK4**	**polo like kinase 4**	0.411	<0.01	0.357	<0.01	0.53	<0.01	1.24	0.277	1.98	<0.001	0.05	0.23
**PPAN**	**peter pan homolog**	0.369	<0.01	0.433	<0.01	-	-	1.09	0.666	-	-	0.313	<0.01
**PPRC1**	**PPARG related coactivator 1**	0.49	<0.01	0.257	<0.01	0.482	<0.01	1.78	0.231	1.38	0.018	0.341	<0.01
**PRMT3**	**protein arginine methyltransferase 3**	0.108	0.2	0.235	<0.01	0.339	<0.01	0.89	0.728	1.06	0.658	0.192	<0.01
**PUS1**	**pseudouridine synthase 1**	0.469	<0.01	0.513	<0.01	0.54	<0.01	1.07	0.819	1.38	0.003	0.146	<0.01
**RABEPK**	**Rab9 effector protein with kelch motifs**	0.129	0.13	0.241	<0.01	0.071	<0.01	0.71	0.383	0.76	0.085	-0.062	0.14
**RCL1**	**RNA terminal phosphate cyclase like 1**	0.099	0.25	0.171	<0.01	0.107	<0.01	1.43	0.368	0.85	0.259	0.302	<0.01
**RRP12**	**ribosomal RNA processing 12 homolog**	0.288	<0.01	0.591	<0.01	0.576	<0.01	1.76	0.152	1.28	0.056	0.173	<0.01
**RRP9**	**ribosomal RNA processing 9, U3 small nucleolar RNA binding protein**	0.496	<0.01	0.565	<0.01	0.453	<0.01	1.09	0.8	1.2	0.393	0.231	<0.01
**SLC19A1**	**solute carrier family 19 member 1**	0.21	0.01	0.58	<0.01	0.631	<0.01	1.37	0.19	1.55	<0.001	0.193	<0.01
**SLC29A2**	**solute carrier family 29 member 2**	0.294	<0.01	0.401	<0.01	-	-	0.91	0.597	-	-	0.267	<0.01
**SORD**	**sorbitol dehydrogenase**	0.096	0.26	0.25	<0.01	0.32	<0.01	1.05	0.797	1.09	0.139	-0.069	0.1
**SRM**	**spermidine synthase**	0.508	<0.01	0.477	<0.01	0.379	<0.01	1.49	0.128	1.16	0.124	0.201	<0.01
**SUPV3L1**	**Suv3 like RNA helicase**	0.514	<0.01	0.428	<0.01	0.426	<0.01	1.47	0.07	1.64	0.002	0.173	<0.01
**TBRG4**	**transforming growth factor beta regulator 4**	-0.228	<0.01	0.66	<0.01	0.517	<0.01	2.01	0.03	1.46	<0.001	0.22	<0.01
**TCOF1**	**treacle ribosome biogenesis factor 1**	0.216	0.01	0.46	<0.01	0.173	<0.01	1.32	0.479	1.3	0.412	0.155	<0.01
**TFB2M**	**transcription factor B2, mitochondrial**	0.291	<0.01	0.227	<0.01	0.161	<0.01	1.61	0.149	0.95	0.554	-0.029	0.49

**TMEM97**	**transmembrane protein 97**	0.382	<0.01	0.499	<0.01	0.562	<0.01	0.76	0.19	1.37	<0.001	0.16	<0.01
**UNG**	**uracil DNA glycosylase**	0.479	<0.01	0.472	<0.01	0.464	<0.01	1.02	0.963	1.3	0.004	0.106	0.01
**UTP20**	**UTP20 small subunit processome component**	0.044	0.61	-0.01	0.81	0.136	<0.01	1.06	0.822	1.12	0.763	0.095	0.02
**WDR43**	**WD repeat domain 43**	0.453	<0.01	0.191	<0.01	0.402	<0.01	1.37	0.42	1.44	0.025	0.31	<0.01
**WDR74**	**WD repeat domain 74**	0.583	<0.01	0.548	<0.01	0.457	<0.01	1.01	0.967	1.35	0.003	0.219	<0.01

Note: For each gene, correlation was performed with proliferation score in all cohorts and R value as well as p value are shown. COX analysis was performed to examine survival difference in TCGA and METABRIC cohorts and hazard ratio (HR) and p value are shown for each gene. Correlation analysis was peformed for each gene using TCGA cohort in order to quantify the positive or negative impact of each target gene on the overall MYC score. Spearman R value and p value for each gene is shown.

Table S3: Baseline patient demographics and clinical characteristics of TCGA cohort

TCGA	**MYC v1**			**MYC v2**		
	**Low**	**High**	***p* value**	**Low**	**High**	***p* value**
	713	352		713	352	
**Age**			0.07			0.056
Median	59	56		59	56	
IQR	49-67	48-67		49-67	48-67	
**Subtype**			<0.001			<0.001
ER+/HER2-	459	120		459	120	
TNBC	106	69		102	73	
HER2+	55	104		52	107	
Unknown	93	59		100	52	
**AJCC**						
**T-category**			<0.001			0.001
T1	209	64		207	66	
T2	386	227		385	228	
T3	95	42		96	41	
T4	22	16		24	14	
Unknown	1	3		1	3	
**N-category**			0.03			0.065
N-	321	183		323	181	
N+	379	161		376	164	
Unknown	13	8		14	7	
**M-category**			1			0.812
M-	587	297		598	286	
M+	13	7		13	7	
Unknown	113	48		102	59	
**Stage**			0.019			0.053
I	135	43		132	46	
II	383	219		384	218	
III	166	76		168	74	
IV	12	6		12	6	
Unknown	17	8		17	8	
**Grade**			<0.001			<0.001
G1	68	8		65	11	
G2	233	28		228	33	
G3	107	123		104	126	
Unknown	305	193		316	182	

Table S4: Baseline patient demographics and clinical characteristics of METABRIC cohort

METABRIC	**MYC v1**			**MYC v2**		
	**Low**	**High**	***p* value**	**Low**	**High**	***p* value**
	1276	628		1276	628	
**Age**			<0.001			<0.001
Median	63	59		63	60	
IQR	54-72	48-69		53-71	49-70	
**Subtype**			<0.001			<0.001
ER+/HER2-	1006	349		1039	316	
TNBC	133	103		122	114	
HER2+	130	168		108	190	
Unknown	7	8		7	8	
**N category**			<0.001			<0.001
N-	711	281		708	284	
N+	565	346		568	343	
Unknown	0	1		0	1	
**Stage**			0.009			<0.001
0	2	2		3	1	
I	345	130		368	107	
II	522	278		509	291	
III	68	47		71	44	
IV	5	4		5	4	
Unknown	334	167		320	181	
**Grade**			<0.001			<0.001
G1	147	18		154	11	
G2	588	152		600	140	
G3	478	449		467	461	
Unknown	63	9		55	16	

Table S5: Baseline patient demographics and clinical characteristics of GSE124647 cohort

GSE124647	**MYC v1**			**MYC v2**		
	**Low**	**High**	***p* value**	**Low**	**High**	***p* value**
	94	46		94	46	
**Metastasis**			0.237			0.569
Local	9	10		12	7	
Lymph node	27	17		29	15	
Bone	10	1		10	1	
Liver	11	5		11	5	
Soft tissue	24	8		22	10	
Other	13	5		10	8	

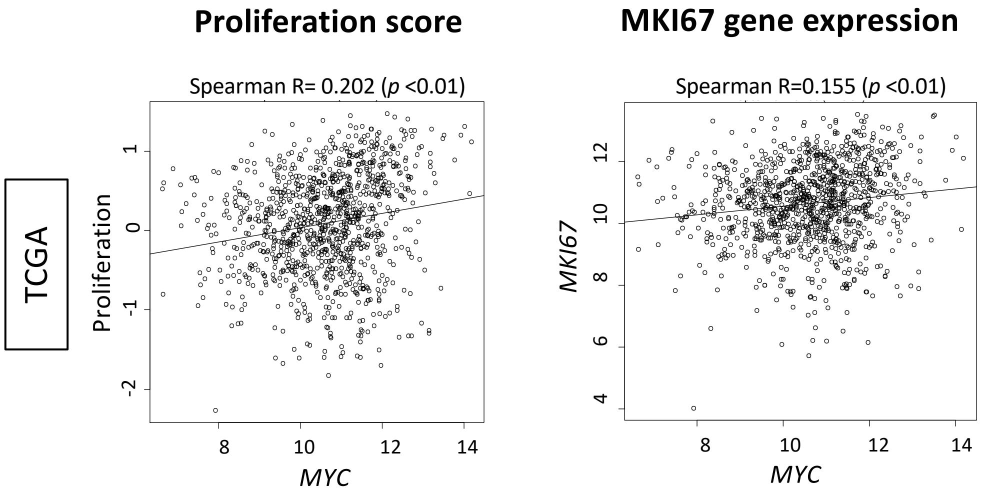

Figure S1: MYC gene expression proliferation. Correlation curve for TCGA cohort demonstrating significant correlation between MYC gene expression and proliferation score as well as MYC gene expression and MKI67 expression.

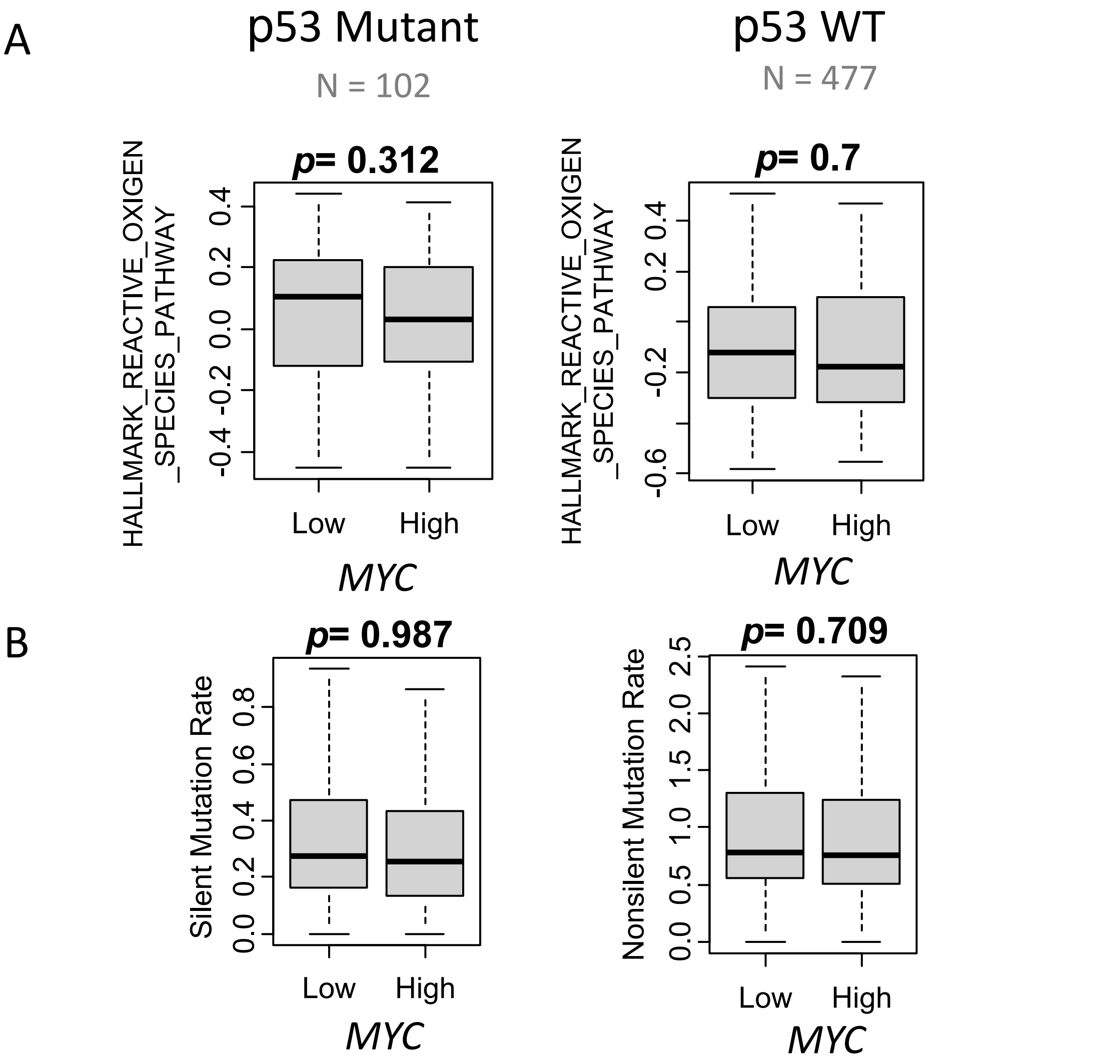

Figure S2: MYC gene expression analysis (ER+/HER2-). TCGA cohort was used to calculate high and low MYC gene expression for ER+/HER2- tumors. **(A)** High levels of gene expression was not significantly associated with increased reactive oxygen species in either p53 wildtype or mutant. **(B)** High levels of MYC expression was not significantly associated with silent or nonsilent mutation rates.
